# Influence of the Nitrogen Content on the Carbide Transformation of AISI M42 High-Speed Steels during Annealing

**DOI:** 10.1038/s41598-018-22461-z

**Published:** 2018-03-12

**Authors:** Yi-Wa Luo, Han-Jie Guo, Xiao-Lin Sun, Jing Guo

**Affiliations:** 1 School of Metallurgical and Ecological Engineering, University of Science and Technology Beijing, Beijing, 100083 P.R. China; 2 Beijing Key Laboratory of Special Melting and Preparation of High-End Metal Materials, Beijing, 100083 P.R. China; 3 Research Institute of High Temperature Materials, Central Iron and Steel Research Institute (CISRI), Beijing, 100081 P.R. China

## Abstract

Attempts were made to elucidate the effect of nitrogen on primary eutectic carbides in as-cast and annealed AISI M42 high-speed steel. Particular emphasis was placed on the transformation of carbides during forging and annealing in steels with different nitrogen concentrations and the influence of final carbides on the impact toughness of the steel. Microstructural observation, electrolytic extraction method, X-ray diffraction analysis, automated inclusion analysis (INCASteel), and impact toughness measurement combined with fractographic observation were conducted on the specimens. Primary M_2_C carbides were found to be dominant precipitates in the as-cast ingot, with a certain amount of V(C,N). Nitrogen addition promoted the formation of fibrous M_2_C, whereas lamellar M_2_C predominated in M42 steel with a low nitrogen concentration (w[N]_%_ = 0.006). Fibrous carbides M_2_C tend to decompose into more stable carbides M_6_C and MC during forging and annealing compared to lamellar M_2_C. Nitrogen alloying only affected the morphologies and dimensions of carbides, but did not change the types of carbides. These improvements in the dimensions and fractions of carbides naturally increased the impact toughness of annealed steel. Hence, it was suggested that the addition of nitrogen to AISI M42 high-speed steel was required to achieve homogeneous distribution of carbides and sufficient impact toughness.

## Introduction

HIGH-SPEED steel (HSS) is one of the most important materials for manufacturing drills, dies, and other cutting tools. Compared with conventional carbon tool steels, cutting tools made of high-speed steel are capable of working at much higher speeds and retaining their hardness^1–3^. The alloy elements in high-speed steel are usually molybdenum, cobalt, tungsten, and vanadium, which all have the ability to form carbides with carbon. These carbides have a beneficial effect on the mechanical properties of high-speed steel, i.e., high hardness and good wear resistance. However, the useful properties greatly depend on the type, quantity, morphology, dimension and distribution of carbides^4–9^. Therefore, a detailed investigation and evaluation of the formation and transformation of carbides in high-speed steels is of primary importance.

AISI M42 high-speed steel is characterized by high contents of molybdenum and cobalt, and the cast structure is usually composed of dendritic and network morphologies of M_2_C-type eutectic carbides. During the heat treatment process, the network of M_2_C carbides will break into smaller and more uniformly distributed carbides and transform into a more stable form, which can be expressed as M_2_C + matrix → M_6_C + MC^10–12^. The final microstructure of the steel consists of matrix and fine carbide precipitates with different orientations.

Nitrogen is known to produce some beneficial effects in high-speed steels. Nitrogen addition promotes precipitation strengthening reactions and solid solution hardening and forms the basis of many high-strength grades^13^. M. Boccalini and H. Goldenstein^14^ indicated that additions of nitrogen increase the supercooling for the eutectic reaction and favour the formation of M_2_C carbide compared to M_6_C carbide. Some researchers have proposed that nitrogen addition promotes the formation of fibrous M_2_C carbide rather than plate-like M_2_C carbide^13,15^. However, systematic research on the effect of nitrogen on the precipitation and subsequent transformation of eutectic carbides in high-speed steel is still deficient.

The aim of this study was to investigate the effect of nitrogen on the characteristics of eutectic carbides in as-cast AISI M42 high-speed steel and to evaluate the transformation of eutectic carbides in annealed steel. This study also identifies the correlation between carbide precipitates and the impact toughness of steel. Two electroslag remelting (ESR) ingots were fabricated with different nitrogen equivalents, and the effects on fracture properties were investigated by observing the fracture surface of annealed steel. Electrolytic extraction method, X-ray diffraction, and automated inclusion analysis (INCASteel) were employed to ascertain the microstructure, composition, dimension and fraction of carbides.

## Experimental Procedures

### Material and Specimen Preparation

Materials used in this study are two AISI M42 HSS ingots manufactured by vacuum induction melting followed by the protective gas electroslag remelting (P-ESR) method, and their chemical compositions are listed in Table 1. The first ESR ingot is the standard AISI M42 HSS, which is denoted as M42. The other one, denoted as M42-N, has been alloyed with nitrogen. In the case of the nitrogen-alloyed steel grade, V-N alloy and ferroalloys (Fe-Cr, Fe-Mn, Fe-W) were used to adjust the chemical compositions. The ESR ingot was 100 mm in diameter and 300 mm in length.

**Table 1:** Chemical Compositions of the AISI M42 HSS (Weight Percent).

No.	C	N	Si	Mn	P	S	Cr	Mo	V	W	Co
M42	1.04	0.006	0.38	0.31	0.011	0.004	4.27	8.45	1.03	1.37	7.35
M42-N	1.06	0.011	0.66	0.40	0.018	0.006	4.57	9.48	1.37	1.70	7.84

An inductively coupled plasma optical emission spectroscopy (ICP-OES) was employed to analyse the contents of alloy elements W, Mn, Co, V, and Mo in the ESR ingots. The nitrogen content was measured by the inert gas fusion-thermal conductivity method.

### Annealing Treatment

The as-cast ESR ingots were forged after being held at 1373 K for 120 min. The specimens used in the following annealing experiment were taken from the forged steel with dimensions of 30 × 30 × 100 mm and annealed in a vacuum induction furnace. The specimens were first heated to 1153 K for 120 min, followed by cooling to 773 K at a rate of 50 K/h. Then the specimens were left in the furnace and cooled to ambient temperature in air.

### Microstructural Analysis

The specimens prepared for the observation of eutectic carbide formation and transformation were taken from the as-cast ESR ingots and annealed steels, respectively. The specimens were diamond polished and examined by a scanning electron microscope (SEM). The types and compositions of the carbides were analysed using energy-dispersive spectroscopy (EDS) and X-ray diffraction analysis. Automated inclusion analysis (INCASteel) was employed to measure the size distribution and volume fraction of the carbide precipitates. INCASteel could detect carbides with a minimum size of 0.587 μm in the range of 10 mm^2^, and measure the equivalent diameter of each carbide in the field of view.

### Carbide Collection and Analysis

In order to identify the three-dimensional morphology and phase compositions of the eutectic carbides, an electrolytic extraction method was performed to collect carbide precipitates in the ESR ingots. A plate sample with dimensions of 20 × 80 × 3 mm was used as a cathode and was electrolyzed in organic solution (1% tetramethylammonium chloride +10% ethylene acetone methanol solution). The total current was controlled to be less than 0.6 A, and the current density was 0.004 to 0.006 A/cm^2^. The temperature of the electrolyte was kept between 0 and −5 °C. After electrolysis, the carbide precipitates were collected and cleaned with 10 g/L citric acid alcohol and distilled water. These dried carbide precipitates were observed using SEM. X-ray diffraction analysis was also employed to identify the phases of the collected carbides.

### Impact Toughness Testing

The impact toughness was obtained for the annealed specimens, whose dimensions were 10 × 10 × 55 mm with a notch, in a JB-W300A test machine. The value of the impact energy is an average of three measurements. Fracture surfaces of the fracture toughness specimens were examined using SEM.

## Results and Discussions

### Precipitate Formation in AISI M42 HSS Calculated Using Thermo-Calc Based on Different Nitrogen Content

**Figure 1:**
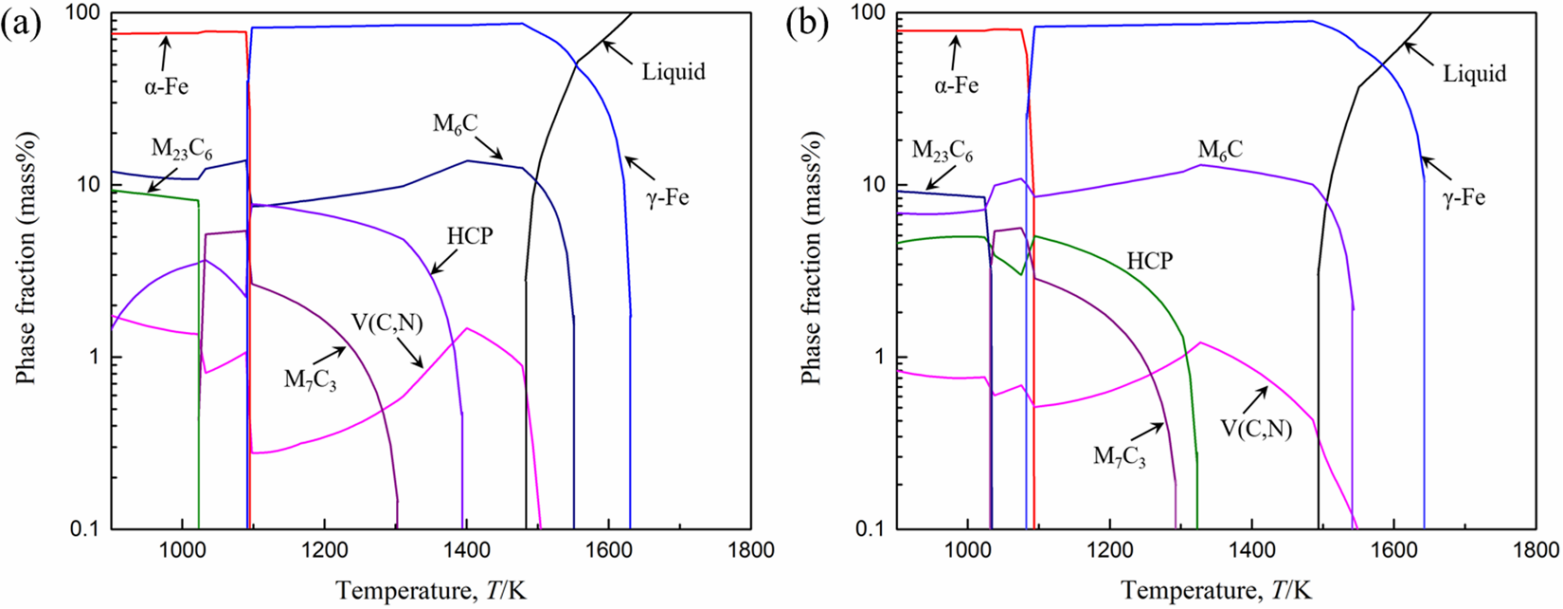
Phase equilibrium diagram of the (**a**) conventional (M42) and (**b**) nitrogen containing (M42-N) AISI M42 HSS calculated using Thermo-Calc software.

**Figure 2:**
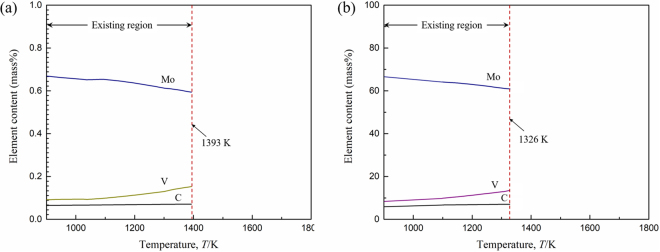
Change in element contents of ‘HCP’ phase in (**a**) conventional (M42) and (**b**) nitrogen containing (M42-N) as a function of temperature.

The types of carbides and carbonitrides present in conventional and nitrogen containing steel were calculated using Thermo-Calc software (TCFE7 database), as shown in Fig. 1. Three different types of carbides were found to precipitate, i.e., M_6_C, M_7_C_3_ and M_23_C_6_, in AISI M42 HSS under the conditions of equilibrium solidification and cooling. First, M_6_C-type carbides precipitate at approximately 1540 K, Whereas, M_7_C_3_ and M_23_C_6_ carbides precipitate successively at 1300 K and 1030 K, respectively (Fig. 1(a)). The type of carbonitrides is mainly V(C,N) because vanadium is the strongest nitride former in AISI M42 HSS. The precipitation temperature of V(C,N) increases with the increase of nitrogen content, while the precipitation temperatures of other carbides are not greatly affected by the change of nitrogen content (Fig. 1(b)). In conventional M42 steel with a nitrogen content of 0.006%, V(C,N) appears at 1500 K after M_6_C carbides. When the nitrogen content increases to 0.011%, V(C,N) precipitates occur preferentially compared to carbide formation with a precipitation temperature of 1550 K. In addition, a kind of HCP phase is also precipitates during the equilibrium solidification of AISI M42 steel. Figure 2 presents the change in the element contents of HCP phase as a function of temperature. According to Fig. 2(a) and (b), HCP phase is a Mo rich close-packed hexagonal structure which composed of a small amount of V and C. Changes in nitrogen contents have little impact on the composition of HCP phase, but they apparently affect the precipitation temperature. In conventional M42 steel with the nitrogen content of 0.006%, HCP phase starts to precipitate at 1393 K (Fig. 2(a)). When the nitrogen content increases to 0.011%, the precipitation temperature decreases to 1326 K (Fig. 2(b)). Consequently, the precipitation temperature of HCP phase decreases with the increasing nitrogen content.

Under the condition of non-equilibrium solidification during production, the AISI M42 HSS has a ledeburitic microstructure that consists of ferrite and large primary M_2_C or M_6_C carbides^6,7,16–18^. The type of carbide is eutectic metastable M_2_C or stable M_6_C, depending on the chemical composition and solidification conditions of the steel.

### Effect of Nitrogen as an Alloying Element on the As-cast Structure

**Figure 3:**
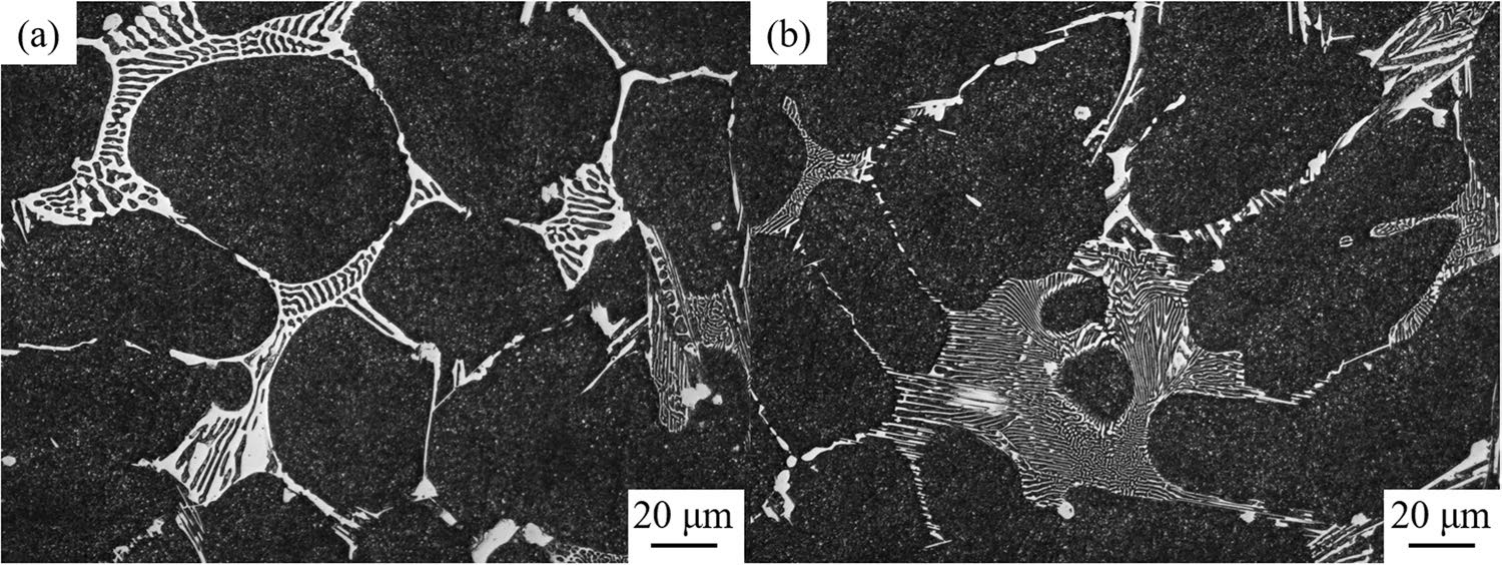
SEM micrograph of the as-cast microstructure of (**a**) M42 and (**b**) M42-N ESR ingots.

A series of backscattered electron images of the microstructure of the ESR ingots with different nitrogen contents are shown in Fig. 3. The as-cast structure of AISI M42 HSS can be divided into two types of constructions: a dendritic matrix of ferrite with small carbides and a ledeburite of carbides and ferrite. A previous study^19^ demonstrated that the smaller carbides were mostly V-rich MC or M(C,N), while the large primary carbides were Mo-rich M_2_C. In conventional M42 steel, the morphology of M_2_C carbides presents a lamellar shape with dimensions of 5 to 20 μm in length and 1 to 2 μm in width (Fig. 3(a)). In the case of nitrogen alloying, the M_2_C carbides become thinner and develop into a fibrous shape. Meanwhile, the distance between the carbides in ledeburite turns narrow (Fig. 3(b)).

Table 2 lists the content of alloying elements in M_2_C carbides of different shapes analysed by EDS. It is found that the content of alloying elements in lamellar M_2_C is more abundant than that in fibrous M_2_C. This means that the alloying elements in the matrix increase with the increase of nitrogen content in the steel.

**Table 2:** Chemical Compositions of M_2_C-Type Carbides (Weight Percent).

M_2_C	Mo	W	Cr	V	Co
lamellar type	30.1	7.4	5.2	6.7	8.2
fibrous type	10.6	3.9	4.4	4.9	6.3

**Figure 4:**
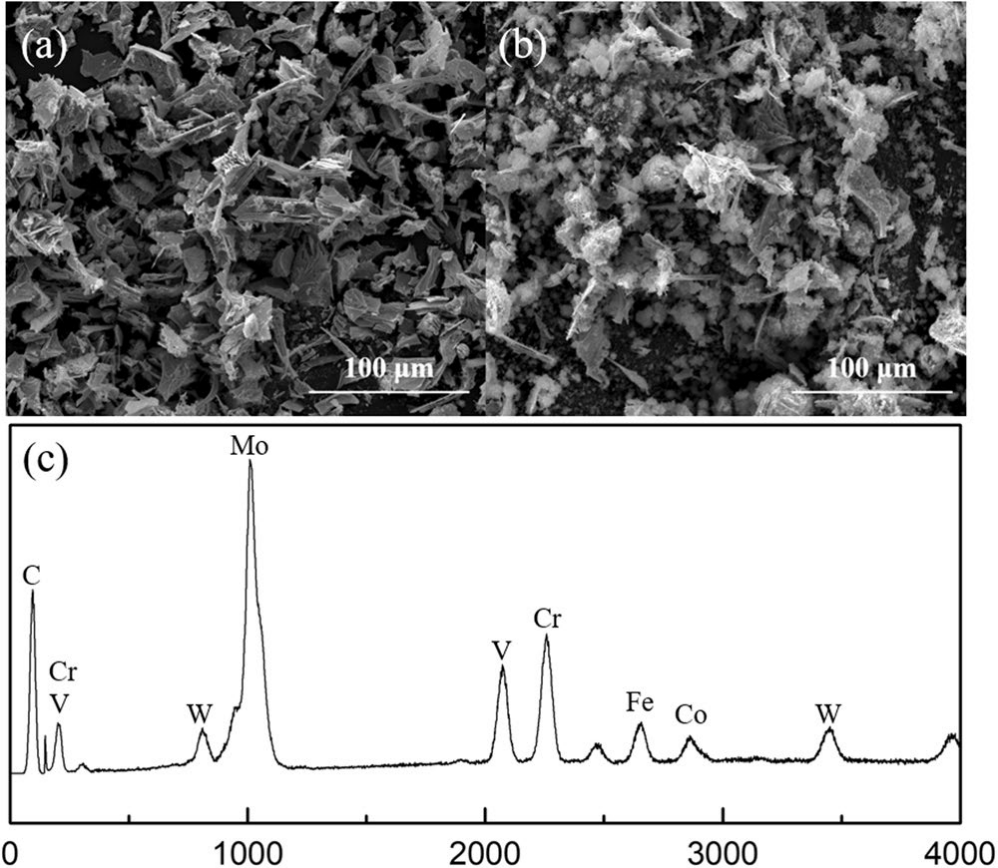
SEM micrograph of carbide precipitates extracted from (**a**) M42 and (**b**) M42-N ESR ingots, and (**c**) EDS spectrum of an observed carbide.

**Figure 5:**
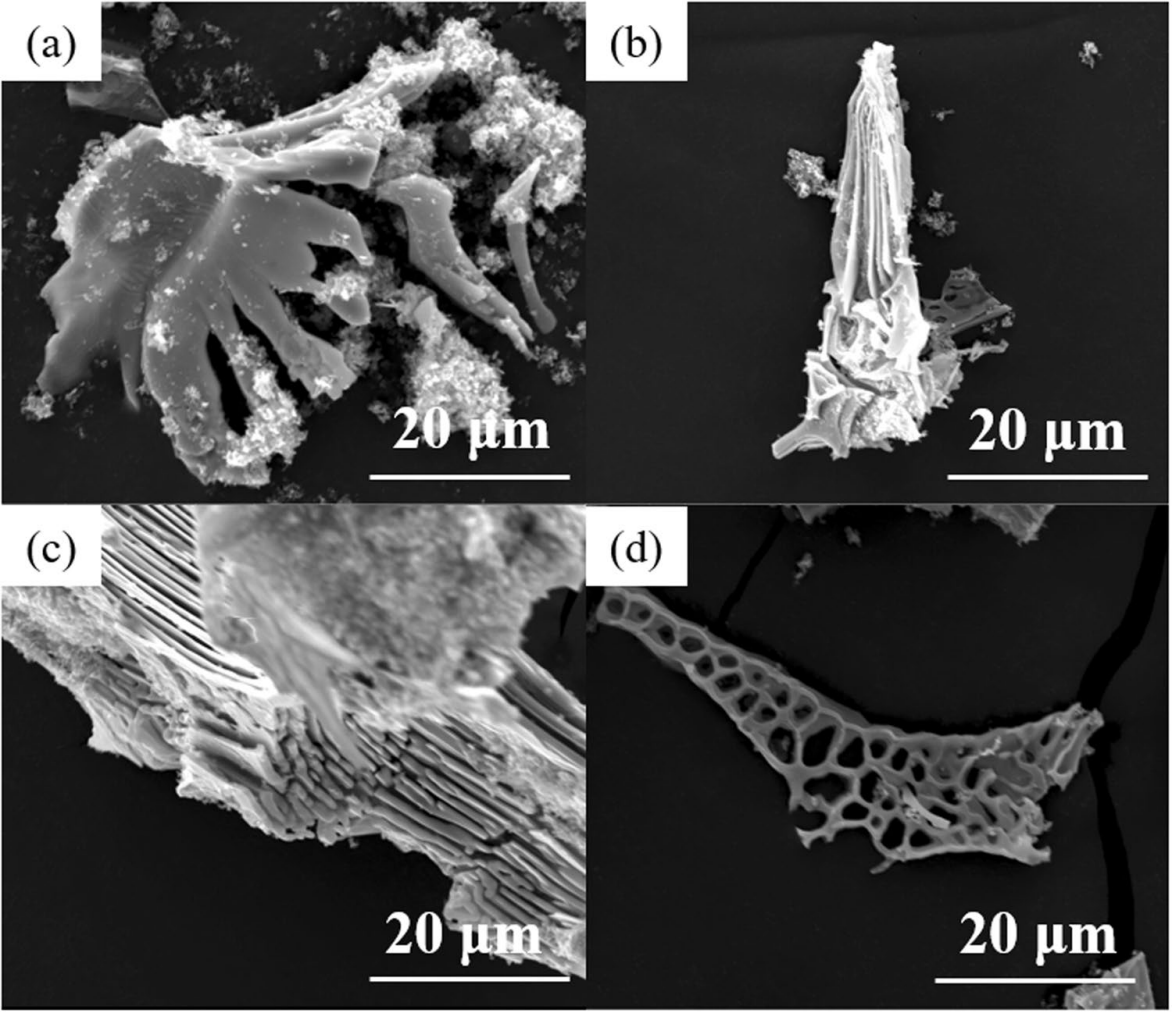
The three-dimensional morphology of typical carbides in (**a**)-(**b**) M42 and (**c**)-(**d)** M42-N ESR ingots.

Figure 4 shows the SEM micrographs and EDS spectrum of the carbides extracted from the conventional and nitrogen-alloyed ESR ingots. The average compositions of the observed carbides analysed by EDS are listed in Table 3. From Fig. 4(a) and (b), it is clear that the morphology of carbides in the conventional M42 as-cast ingot presents a distinct difference from that of carbides after nitrogen treatment. Carbides electrolytically extracted from conventional ESR specimens are mainly angular polyhedrons, which easily give rise to stress concentration. With the increase of nitrogen content in the steel, both the morphology and the dimension of carbide precipitates have been modified to a large extent. Figure 5 shows the three-dimensional morphology of typical individual carbides observed in the specimens. Coarse carbides are extracted in bulky form from conventional ESR ingots (Fig. 5(a) and (b)). In contrast, the carbides in nitrogen-alloyed steel present fibrous or honeycomb morphology (Fig. 5(c) and (d)).

**Table 3:** Average Compositions of the Carbides in ESR Ingots Determined by EDS (Weight Percent).

No.	C	Mo	W	Cr	Co	V	Fe
M42	15.1	60.4	5.3	7.9	1.6	7.2	2.5
M42-N	19.5	57.7	4.2	5.1	1.0	7.2	5.3

According to EDS analysis, as shown in Fig. 4(c) and Table 3, the observed carbides contain mainly Mo, with a trace amount of Cr, V, Co, W, and Fe atoms. There is no palpable difference in the compositions of alloying elements in the carbides between conventional and nitrogen alloyed ESR ingots. However, the concentrations of alloying elements in the carbides are higher in specimen M42 compared with specimen M42-N. This phenomenon is in reasonable agreement with the measurement result in Table 2. As a consequence, increasing the nitrogen content promotes the optimization of the morphology of carbides and decreases the concentration of alloying elements in carbides.

**Figure 6:**
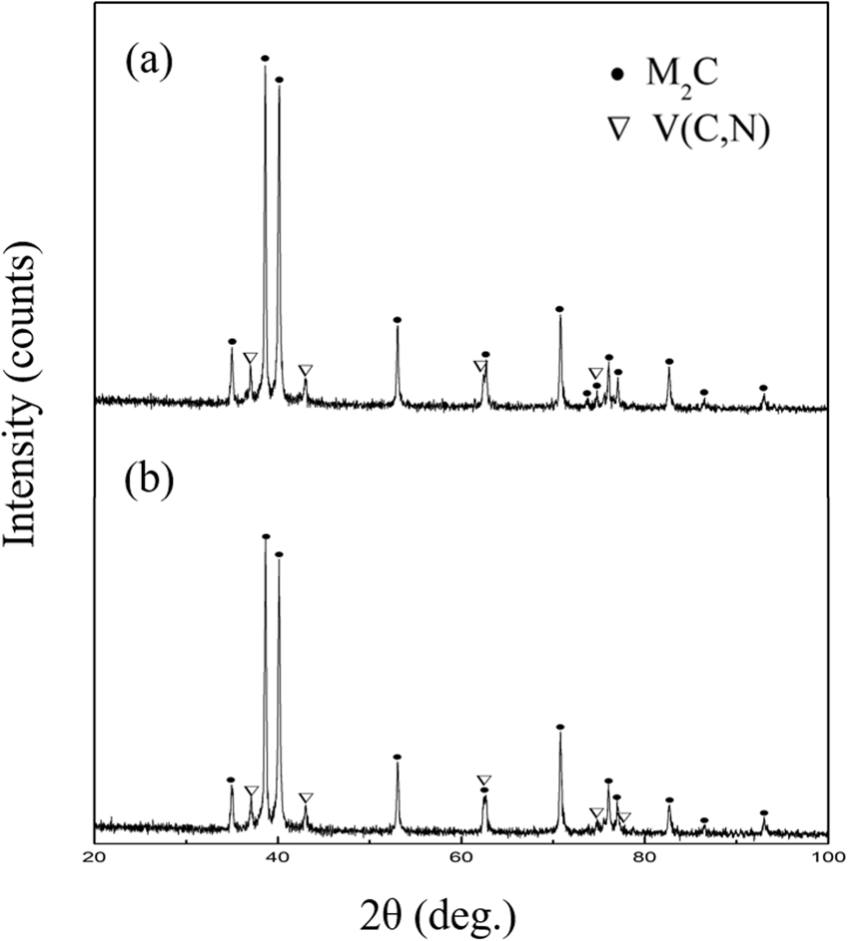
X-ray diffraction patterns of the carbide precipitates extracted from (**a**) M42 and (**b**) M42-N ESR ingots. Showing peaks of M_2_C and V(C,N).

The X-ray diffraction analysis data of the carbide precipitations are shown in Fig. 6(a) and (b). The presence of M_2_C and V(C,N) is detected in both carbide precipitates. This result indicated that nitrogen addition does not change the type of carbides in the ESR ingot, as confirmed by SEM and EDS research.

### Effect of Nitrogen on the Carbide Transformation and Impact Toughness of Annealed Steel

M_2_C-type carbide is metastable and decomposes into M_6_C and MC during forging and annealing of the steel^20–22^. Compared with M_6_C, MC is rich in vanadium, the amount of which depends on the vanadium concentration in high-speed steel. It is important to point out that V-rich MC usually exists in composite carbonitride V(C,N), rather than the monocrystalline carbide in nitrogen-containing steels. Meanwhile, VC and V(C,N) have the same crystal structure, and the lattice constants are very close. Accordingly, the V-rich MC obtained by carbide transformation was also regarded as V(C,N) in this study, as X-ray diffraction determination of the precipitates was based on their crystallography and not their composition.

**Figure 7:**
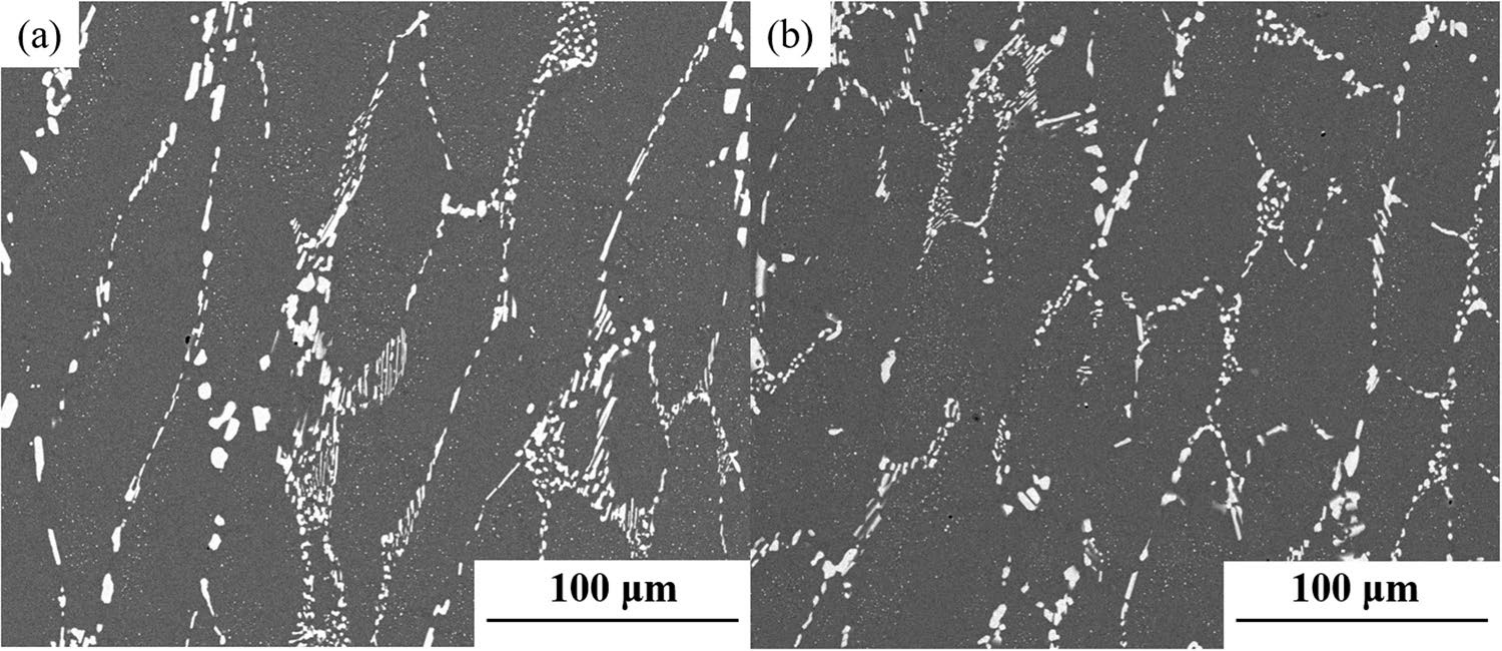
SEM micrograph of the microstructure of (**a**) M42 and (**b**) M42-N HSS after annealing.

**Figure 8:**
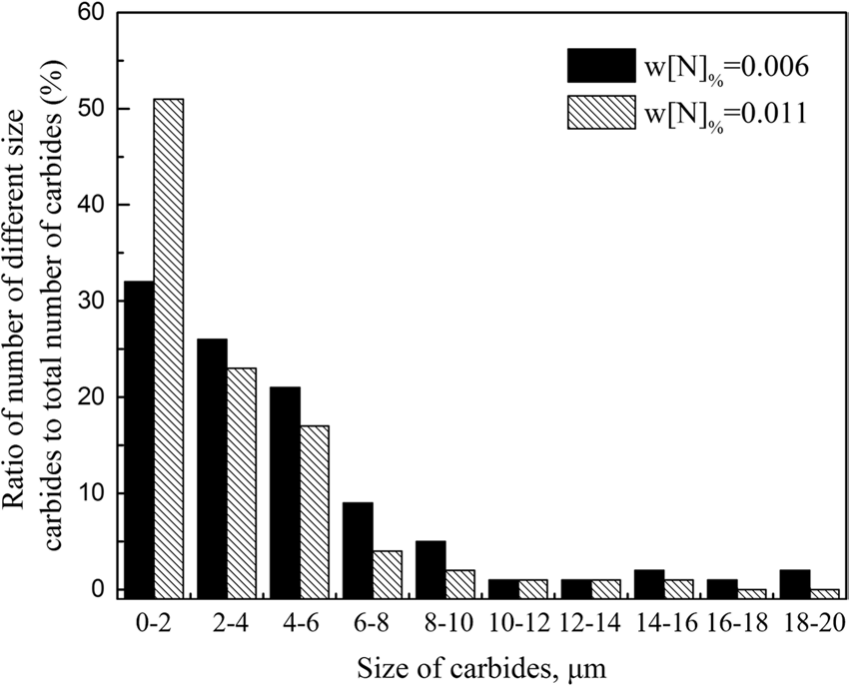
Effect of nitrogen components on the dimension of carbides in AISI M42 HSS after annealing.

The microstructures of the M42 and M42-N specimens after forging and annealing treatment are shown in Fig. 7. It is clear that the continuous network distribution of eutectic carbides changed into a broken network in each annealed specimen. The ratios of the number of carbides of different sizes to the total number of carbides in both specimens were evaluated by INCASteel and shown in Fig. 8. In conventional M42 steel, 32 percent of carbides in total precipitates are less than 2 μm in size, and the ratio decreases with the increase of the dimension of statistical carbides. Moreover, carbides larger than 14 μm also account for approximately 5 percent. In the case of nitrogen alloying, the proportion of carbides smaller than 2 μm shows an increase of 20 percent over conventional M42 steel. More than 90 percent of carbides are less than 10 μm. The statistical results indicate that fibrous M_2_C carbides more easily decompose into small and uniform carbides M_6_C and MC.

**Figure 9:**
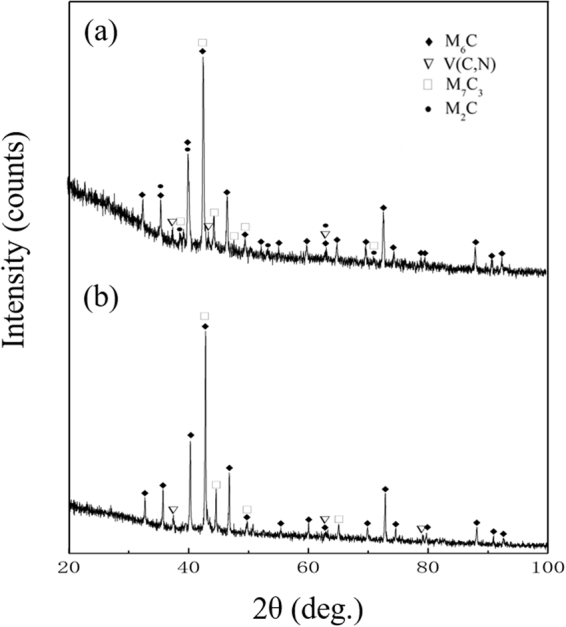
X-ray diffraction patterns of the carbide precipitates extracted from (**a**) M42 and (**b**) M42-N HSS after annealing. Showing peaks of M_2_C, M_6_C, M_7_C_3_, and V(C,N).

In order to identify the type of carbide in both specimens, the carbide precipitates were electrolytically isolated from the steel matrix and analysed by X-ray diffraction. Figure 9 shows the X-ray diffraction patterns of the carbides in each specimen. The presence of M_6_C, V(C,N), and M_7_C_3_ was detected in M42 and M42-N specimens after annealing, while M_2_C-type carbides were also detected in conventional high-speed steel. This phenomenon could be explained by the fact that the carbide transformation (M_2_C + matrix → M_6_C + MC) starts at the interface between the matrix and the M_2_C particles, and moves towards the centre of the carbide^18,23^. Hence, the lamellar carbides decompose incompletely, and M_2_C remains in the centre of the carbide particles. This proves that the thermal stability of lamellar M_2_C is stronger than that of fibrous M_2_C, which is confirmed by the SEM and INCASteel research (Figs 7 and 8).

The carbide precipitates were classified by type through the fractionation method and evaluated by ICP-OES. To separate different types of carbides, the carbide powder was insulated in aqueous solution (6% H_2_SO_4_ + 20% H_2_O_2_ + 1% citric acid) in boiling water bath until M_6_C-type carbides and V(C,N) were completely dissolved. The M_7_C_3_-type carbides were insoluble in the aqueous solution and separated from the precipitated powder. The average compositions of M_7_C_3_-type carbides and M_6_C-type carbides in conventional and nitrogen containing specimens are shown in Tables 4 and 5. The compositions of M_6_C also contain V(C,N), because the chemical properties of these two kinds of precipitates are similar, they are difficult to separate by fractionation. As shown in Table 4, there is no perceptible difference in the concentrations of alloying elements in M_7_C_3_-type carbides in different specimens. M_7_C_3_ contains mainly Cr and Fe, with minor amounts of Co, Mo, and W atoms. M_6_C carbides are Mo-rich carbides containing Fe and a trace amount of Cr, W, and Co (Table 5). It is worth noting that N prefers to exist as precipitates rather than as a solid solution in conventional M42 steel or nitrogen-alloyed M42 steel.

**Table 4:** Average Compositions of M_7_C_3_-Type Carbides in M42 HSS after Annealing.

M_7_C_3_	Element (mass%)							Formula
	Fe	Cr	Co	Mo	W	C	Σ	
M42	1.559	2.498	0.071	0.209	0.013	0.416	4.766	(Fe_0.336_Cr_0.577_Co_0.009_Mo_0.026_W_0.001_)_7_C_3_
M42-N	1.753	2.544	0.052	0.310	0.025	0.465	5.149	(Fe_0.347_Cr_0.541_Co_0.010_Mo_0.036_W_0.002_)_7_C_3_

**Table 5:** Average Compositions of V(C,N) and M_6_C-Type Carbides in M42 HSS after Annealing.

V(C,N) + M_6_C	Element (mass%)							
	Fe	Cr	Co	Mo	W	V	N	Σ
M42	3.381	0.764	0.536	5.501	1.331	0.987	0.005	12.505
M42-N	4.394	0.843	0.659	8.694	1.401	0.998	0.011	17.000

**Figure 10:**
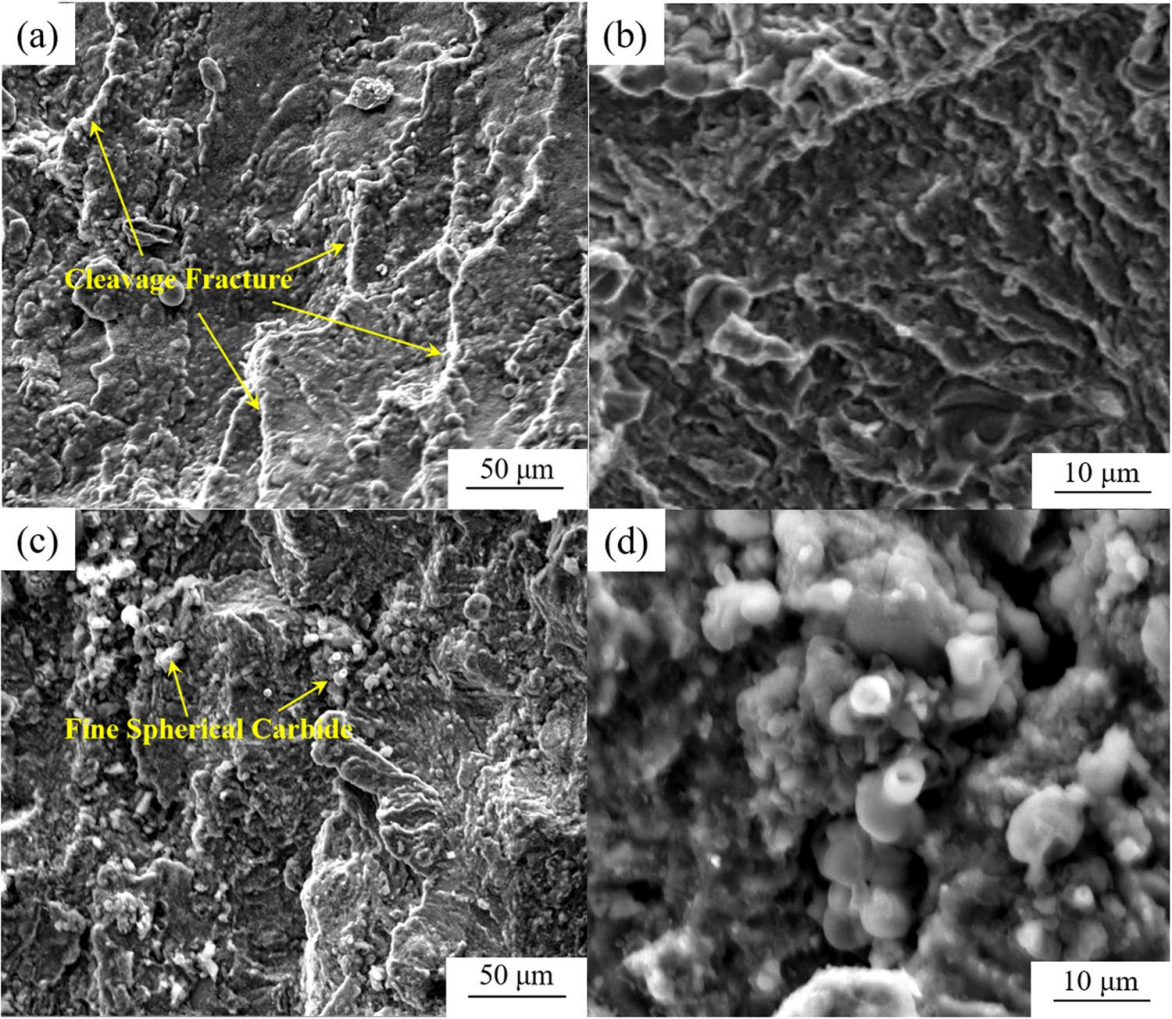
SEM fractography of the impact toughness specimens of the (**a**) annealed M42, (×1000), (**b**) annealed M42, (×5000), (**c**) annealed M42-N (×1000), and (**d**) annealed M42-N (×5000) HSS.

The impact energies of conventional and nitrogen containing M42 steels after annealing are 8.1 ± 0.2 J and 12.6 ± 0.6 J, respectively. The increase in the amount of nitrogen was confirmed to improve the impact toughness of the steel. Figure 10 shows the fractography of AISI M42 high-speed steel specimens with different nitrogen contents after forging and annealing. On the fracture surface of conventional M42 steel, the cleavage fracture mode is predominant, and the ductile facture mode is hardly found (Fig. 10(a)). A number of large primary carbides are pulled out or broken to form cleavage fracture facets (Fig. 10(b)). In the case of nitrogen-alloyed M42 steel, fine spherical carbides were observed to locate at the fracture surface, as shown in Fig. 10(c). Spherical carbides were presented as cluster due to higher concentration of alloying elements at these regions (Fig. 10(d)). Moreover, the signs of plastic deformation on the fracture surface of nitrogen-alloyed M42 steel are much more remarkable. Because most coarse carbides in high-speed steels are located along the cell boundaries and are much harder than the matrix, microcracks initiate along the crystal boundaries and are facilitated by these coarse carbides^24–27^. The size, fraction and distribution of carbides located in the intercellular region determine the fracture behaviour of the steel. Consequently, impact toughness could be enhanced when the amount of carbides is smaller and more uniform. It is confirmed from Figs 7 and 8 that impact toughness increases as the volume fraction of fine carbides increases.

## Conclusions

In this study, the effects of nitrogen on the microstructure, primary carbides, and toughness properties of AISI M42 high-speed steel were investigated. The main conclusions are summarized as follows:

1. The as-cast high-speed steel AISI M42 consisted of unstable primary and eutectic carbides M_2_C and carbonitride V(C,N) in the matrix. The structure of M_2_C carbide is very sensitive to the nitrogen content of high-speed steel. M_2_C presents a lamellar shape in steel with a low nitrogen content of 0.006%, whereas a fibrous shape is observed as the nitrogen content increases to 0.011%.
2. Forging and annealing resulted in a partial transformation of the lamellar M_2_C carbides into small and stable carbides of M_6_C and MC, with an associated change in the crystal orientation. This carbide transformation could be more complete for fibrous M_2_C in the nitrogen-containing (w[N]_%_ = 0.01) M42 steel.
3. Increasing nitrogen content in AISI M42 high-speed steel improves both carbide morphology and dimensions but does not change the types of carbides. Controlling eutectic M_2_C carbides to have a fibrous shape by nitrogen alloying of as-cast ingots is propitious to preventing large undecomposed residual eutectic carbides after annealing and increases the impact energies of annealed steel accordingly.
4. According to the apparent impact toughness research, toughness was determined by the dimension and distribution of carbides. High impact toughness derives from the fine uniform carbides that are refined by nitrogen.

### Data availability statement

All data generated or analysed during this study are included in this published article (and its Supplementary Information files).
